# Comparison of six phantoms for entrance skin dose evaluation in 11 standard X‐ray examinations

**DOI:** 10.1120/jacmp.v6i1.2020

**Published:** 2005-03-17

**Authors:** Gaetano Compagnone, Laura Pagan, Carlo Bergamini

**Affiliations:** ^1^ Medical Physics Department S. Orsola‐Malpighi Hospital Via Massarenti 9 40138 Bologna Italy

**Keywords:** anthropomorphic phantom, diagnostic radiology, entrance dose measurements, entrance skin exposure, phantom study

## Abstract

Entrance skin dose (ESD) is an important parameter for assessing the dose received by a patient in a single radiographic exposure. The most useful way to evaluate ESD is either by direct measurement on phantoms using an ionization chamber or using calculations based on a mathematical model. We compared six phantoms (three anthropomorphic, two physical, and one mathematical) in 11 standard clinical examinations (anterior‐posterior (AP) abdomen, posterior‐anterior (PA) chest, AP chest, lateral (LAT) chest, AP lumbar spine, LAT lumbar spine, LAT lumbo‐sacral joint, AP pelvis, PA skull, LAT skull, and AP urinary tract) for two reasons: to determine the conversion factors to use for ESDs measured on different phantoms and to validate the mathematical model used. First, a comparison was done between the three anthropomorphic phantoms (Alderson Rando, chest RSD‐77SPL, and 3M skull) and the two physical phantoms (Uniform and AAPM 31); for each examination we obtained “relative entrance skin dose factors.” Second, we compared these five phantoms with the mathematical phantom: the overall accuracy of the model was better than 14%. Total mathematical model and total ionization chamber uncertainties, calculated by quadratic propagation of errors of the single components, were estimated to be on the order of ±12% and ±3%, respectively. To reduce the most significant source of uncertainty, the overall accuracy of the model was recalculated using new backscatter factors. The overall accuracy of the model improved: better than 12%. For each examination an anthropomorphic phantom was considered as the gold standard relative to the physical phantoms. In this way, it was possible to analyze the variations in phantom design and characteristics. Finally, the mathematical model was validated by more than 400 measurements taken on different phantoms and using a variety of radiological equipment. We conclude that the mathematical model can be used satisfactorily in ESD evaluations because it optimizes available resources, it is based on direct measurements, and it is an easy dynamic tool.

PACS number(s): 87.66.Xa

## I. INTRODUCTION

Entrance skin dose (ESD) is an important parameter in assessing the dose received by a patient in a single radiographic exposure. The European Union has identified this physical quantity as one to be monitored as a diagnostic reference level in the hopes of optimizing patient dose.^(1)^ ^,^ ^(2)^ It is possible to evaluate ESD either by direct measurements (on suitable phantoms using ionization chambers or on patients using thermoluminescent dosimeters, TLDs) or using mathematical model calculations based on the X‐ray tube output.^(1)^ Using TLDs is time‐consuming in large hospitals. Therefore, in this paper ESDs were evaluated using both measurements taken by ionization chambers and values calculated by a mathematical model; this allowed us to study the accuracies inherent in different experimental setups. To evaluate the ESD, it is necessary to use “standard phantoms,”^(1)^ ^,^ ^(3)^ and it is important to know the difference between them because some may be bought commercially or “home‐made.”^(4)^ ^,^ ^(5)^ There is no advice in the literature to help with phantom selection in different clinical situations.^(6)^ Therefore, two or more similar phantoms are often available in medical physics departments for dosimetric measurements in conventional radiology. Moreover, these phantoms are not always available simultaneously (for instance, one phantom may be being used by someone else); it is also necessary to have conversion factors between different phantoms.

This paper reports a comparison between ESDs measured by five phantoms in 11 standard clinical examinations (anterior‐posterior (AP) abdomen, posterior‐anterior (PA) chest, AP chest, lateral (LAT) chest, AP lumbar spine, LAT lumbar spine, LAT lumbo‐sacral joint, AP pelvis, PA skull, LAT skull, and AP urinary tract) in order to have “relative ESD factors” (REFs) between each phantom and the others. These REFs can be used in a normal dosimetry routine where the ESD measurements have been done using different phantoms for the same kind of examination, performed either after long time intervals with the same radiological system (one X‐ray tube and one generator in a well‐identified radiological room) or using similar clinical technique factors with different radiological equipments (different X‐ray tubes and generators but similar types of radiological apparatus). In addition, a comparison with ESDs calculated by a mathematical model (which can be considered as a sixth phantom) is made, because this is another possibility—easier but less accurate—for this kind of evaluation.

## II. MATERIALS AND METHODS

As explained by Moores,^(7)^ the phantoms for dose assessment can be anthropomorphic (they possess aspects of anatomical structure), physical (they do not attempt to reproduce anatomical details directly and may range from a single block of material to more sophisticated structures), or mathematical (they may be simple mathematical models to represent the interaction of X‐ray beams with biological tissue in order to assess ESD or other dosimetric parameters). In this study, we first compared three anthropomorphic and two physical phantoms. Then these five phantoms were compared with one mathematical phantom, as described below.

### A. Anthropomorphic and physical phantom comparison

The standard examinations, clinical technique factors used (with respective standard deviations), and number of both radiographic systems and measurements considered in this study are shown in Table 1. The technique factors reported for each examination are the following: the average kilovolt peak (kVp), milliampere x seconds (mAs), and focus‐to‐phantom surface distance (FSD) values used clinically in our hospital. Nearly all the X‐ray generators were three‐phase (6 or 12 pulse) models or high‐frequency generators. All radiographic systems were controlled by an ISO 9001–2000 certified quality assurance program, which provides good equipment performance according to acceptance, status, and constancy tests. In particular, to control the reproducibility and the linearity of the X‐ray tube output, three measurements free‐in‐air at two different kVp values and at five different mAs settings (total: 30 output measurements, 15 at 80 kVp and 15 at 100 kVp, for each X‐ray tube) were taken. These measurements were taken yearly or when a tube was replaced. The acceptability limit for both reproducibility and linearity was 10%.

**Table 1:** The 11 examinations, averages, and standard deviations of technique factors, number of radiological systems, and number of measurements considered in this study

Examination	kVp	mAs	FSD (cm)	Number of radiological systems	Number of measurements
AP abdomen	77±10	32±31	87±9	25	51
PA chest	101±25	9±7	170±16	12	29
AP chest	85±15	5±4	104±14	12	37
LAT chest	107±20	16±15	148±30	12	28
AP lumbar spine	72±5	52±32	87±8	16	35
LAT lumbar spine	86±5	90±77	88±8	16	30
LAT lumbo‐sacral joint	85±7	65±56	87±7	13	26
AP pelvis	74±10	34±32	88±10	26	48
PA skull	72±8	30±19	90±8	18	40
LAT skull	66±7	32±23	91±8	17	40
AP urinary tract	78±9	36±33	86±10	20	44

**Figure 1:**
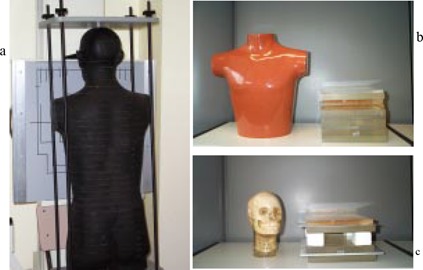
The five anthropomorphic and physical phantoms used in this study: (a) Alderson Rando; (b) chest RSD−77SPL and uniform; (c) skull 3M and AAPM31.

The direct ESD measurements were made on five different phantoms, the first three anthropomorphic (Fig. 1): Alderson Rando (ALD); chest RSD‐77SPL (CHE), Radiology Support Device, Long Beach, USA; skull 3M (SKU); uniform (UNI), 25cm×25cm×20cm polymethyl methacrylate (PMMA); American Association of Physicists in Medicine (AAPM) phantom as described in AAPM Report No. 31(A31),^(8)^ which combines PMMA with aluminum sheets and air gaps, in order to simulate various anatomical parts.

For each measurement, the phantoms were positioned as a standard patient, and for each phantom the same technical parameters (kVp, mAs, FSD, and X‐ray field size as indicated by senior radiologists) were selected. The ESDs were measured as indicated in other papers,^(9)^ by positioning at the surface of each phantom on the beam central axis an ionization chamber model 90X6–6 connected to a Radiation Monitor Controller model 9010 (Radcal Corporation, Monrovia, CA).

The experimental setup was chosen so that the variables possibly affecting the results were carefully controlled: the detector did not significantly perturb the photon fluence on the phantom surface underneath it; the cross‐sectional area of the detector was significantly less than the area of the irradiated phantom; and the FSD was measured by taking into account the position of the focal spot inside the X‐ray tube.

All instruments are calibrated yearly, with the calibration traceable to an SIT (National Calibration Service in Italy) center. To verify kVp accuracy, direct measurements during the exposures were taken with a noninvasive kVp meter model Mult‐O‐Meter 510 (Unfors Instruments, Billdal, Sweden).

### B. Mathematical versus anthropomorphic and physical phantom comparison

Every ESD measured on anthropomorphic and physical phantoms was compared with the corresponding ESD calculated with the mathematical phantom.

To determine the output *K* of a diagnostic X‐ray tube (in terms of absorbed dose to air or exposure free‐in‐air), many mathematical models have been suggested.^(^ ^10^ ^–^ ^14^ ^)^ In this study, the model proposed by Harpen^(14)^ was adopted:

(1) $$K(\text{kVp},\text{mAs})=\alpha {\left(\text{kVp}\right)}^{\beta }\text{mAs}$$

where parameters α and β depend on the type of X‐ray generator, anode material, FSD, and X‐ray tube filtration.

Equation (1) gives *K* as a function of kVp and mAs, by taking only two X‐ray tube output measurements at two different voltages. To this end, the average of the 15 measurements taken at 80 kVp and the average of the 15 measurements taken at 100 kVp during the reproducibility and linearity quality controls mentioned above were used as the two output values.

Harpen's formula gives the absorbed dose to air, free‐in‐air; therefore, to determine ESD, some corrections must be made for backscatter factors (BSF). European guidelines^(1)^ propose a simple and generic value for conventional radiography: 1.35.

For each phantom *j* and examination *k*, the integral accuracy $A_{\text{phantom}j}$ of the mathematical model relative to a single phantom and the differential accuracy $A_{\text{singlepoint}}$ relative to a single measurement were calculated. The $A_{\text{phantom}j}$ relative to examination *k* is defined to be

(2) $$A_{\text{phantom}\;j}=\frac{1}{N_{j}}{\sum_{i=1}^{N_{j}}\left(\frac{\left|\text{MOD}‐\text{PHA}\right|}{\text{PHA}}\right)}_{i}$$

where $N_{j}$ is the number of measurements made with the phantom *j* in each examination *k*, MOD is the ESD value calculated by the mathematical model, and PHA is the ESD value measured on the phantom *j*. In this way, it was possible to calculate $A_{\text{ALD}},A_{\text{CHE}},A_{\text{SKU}},A_{\text{UNI}}$, and $A_{A31}$ for each specific phantom and examination.

The $A_{\text{singlepoint}}$ is defined by the equation

(3) $$A_{\text{singlepoint}}=\frac{\text{MOD}‐\text{PHA}}{\text{PHA}}$$

This is analogous to but slightly different than Eq. (2): the numerator of Eq. (2) uses the absolute value of the difference in the ESDs, whereas the numerator in Eq. (3) does not.

To achieve better accuracy of the mathematical model, for each examination every MOD value was recalculated using the more accurate BSF values given in Harrison^(15)^ and Grosswendt^(16)^ (see Table 2 for examples of typical values used), obtaining new integral accuracies ${}^{\text{NEW}}A_{\text{ALD}},{}^{\text{NEW}}A_{\text{CHE}},{}^{\text{NEW}}A_{\text{SKU}},{}^{\text{NEW}}A_{\text{UNI}}$, and ${}^{\text{NEW}}A_{31}$ for each phantom.

**Table 2:** Typical measured values of ESDs at different phantoms, values obtained from the mathematical model and typical α, β, and backscatter factors values

Examination	Phantom	ESD measured (mGy)	ESD mathematical model (mGy)	α(x1000)	β	BSF
AP abdomen	ALD	3.00	2.29	9.849	1.988	1.35
PA chest	CHE	0.20	0.25	2.580	2.016	1.47
AP chest	CHE	0.36	0.32	6.893	2.016	1.35
LAT chest	CHE	0.88	0.80	3.404	2.016	1.47
AP lumbar spine	ALD	2.98	3.29	10.478	2.042	1.32
LAT lumbar spine	ALD	7.41	8.11	10.241	2.040	1.35
LAT lumbo‐sacral joint	ALD	8.30	7.42	12.256	2.062	1.35
AP pelvis	ALD	2.66	2.84	9.627	2.084	1.32
PA skull	SKU	1.91	1.73	9.204	2.006	1.29
LAT skull	SKU	1.45	1.52	9.002	2.006	1.29
AP urinary tract	ALD	2.50	2.79	10.066	2.006	1.35

The “overall accuracy of the mathematical model” $A_{\text{OVE}}$ relative to all the phantoms and all the examinations was also calculated. It is defined to be

(4) $$A_{\text{OVE}}=\frac{1}{5}\sum_{j=1}^{5}\left(\frac{1}{N_{j\text{TOT}}}\right)\sum_{k=1}^{M}{\left(A_{\text{phantom}\;j}N_{j}\right)}_{k}$$

where $N_{j\text{TOT}}$ is the number of measurements made with the phantom *j* in all examinations, and *M* is the number of examinations in which the phantom *j* was used. When the more accurate BSF values are used, a new overall accuracy ${}^{\text{NEW}}A_{\text{OVE}}$ is obtained.

## III. RESULTS

All the factors presented in this study are “relative” values. Therefore, to obtain a general idea and a comparison of the “absolute” values, consider Table 2. The typical measured values of ESDs at different typical anthropomorphic phantoms, the values obtained from the mathematical model (using the BSF values shown), and typical values of α and β of Eq. (1) are listed in Table 2 based on values in Table 1. The value of *K* depends linearly on the α value, which is related to a clinical technique, in particular, to the inverse of FSD, and exponentially on parameter β, which is more sensitive to intermachine variations and has almost a constant value.

The mean values of REFs measured on anthropomorphic and physical phantoms are given in Table 3; they too are based on Table 1. For each measurement the ratio between the ESD measured on Phantom1 to the ESD measured on the Phantom2 (in the columns Phantom1/Phantom2) was calculated. For each examination, the averages of the ratios taken with all radiological systems used are shown in Table 3. When a phantom is not appropriate for an examination (e.g., a chest phantom for an AP abdomen examination), N/A is indicated.

**Table 3:** REF (relative ESD factors). The factors are the averages of the ratios of the ESDs measured on different phantoms based on Table 1.

		Relative ESD factors		
(a) Alderson Rando phantom Examination	ALD/CHE	ALD/SKU	ALD/UNI	ALD/A31
AP abdomen	N/A	N/A	0.95	1.03
PA chest	1.09	N/A	0.96	1.04
AP chest	1.15	N/A	0.92	1.03
LAT chest	1.19	N/A	1	1.02
AP lumbar spine	N/A	N/A	1.09	0.96
LAT lumbar spine	N/A	N/A	1.56	1.44
LAT lumbo‐sacral joint	N/A	N/A	1.15	1.08
AP pelvis	N/A	N/A	1.20	1.02
PA skull	N/A	1.01	0.85	0.92
LAT skull	N/A	1.01	0.89	0.97
AP urinary tract	N/A	N/A	1.20	1.02

(b) Chest phantom Examination	CHE/ALD	Relative ESD factors CHE/UNI	CHE/A31
PA chest	0.92	0.83	0.95
AP chest	0.87	0.67	0.88
LAT chest	1.10	0.90	0.87

(c) Skull phantom Examination	SKU/ALD	Relative ESD factors SKU/UNI	SKU/A31
PA skull	0.99	0.83	0.92
LAT skull	0.99	0.88	0.95

		Relative ESD factors		
(d) Uniform phantom Examination	UNI/ALD	UNI/CHE	UNI/SKU	UNI/A31
AP abdomen	1.05	N/A	N/A	0.94
PA chest	1.04	1.22	N/A	1.09
AP chest	1.09	1.58	N/A	0.87
LAT chest	1.22	1.12	N/A	1.02
AP lumbar spine	0.94	N/A	N/A	0.90
LAT lumbar spine	0.74	N/A	N/A	0.95
LAT lumbo‐sacral joint	0.89	N/A	N/A	0.95
AP pelvis	0.90	N/A	N/A	1.02
PA skull	1.18	N/A	1.21	1.09
LAT skull	1.12	N/A	1.14	1.08
AP urinary tract	0.90	N/A	N/A	0.92

		Relative ESD factors		
(e) AAPM31 phantom Examination	A31/ALD	A31/CHE	A31/SKU	A31/UNI
AP abdomen	1	N/A	N/A	1.12
PA chest	0.96	1.05	N/A	0.92
AP chest	0.97	1.15	N/A	1.25
LAT chest	0.98	1.15	N/A	0.98
AP lumbar spine	1.04	N/A	N/A	1.14
LAT lumbar spine	0.77	N/A	N/A	1.07
LAT lumbo‐sacral joint	0.94	N/A	N/A	1.07
AP pelvis	1.01	N/A	N/A	1.06
PA skull	1.10	N/A	1.09	0.92
LAT skull	1.03	N/A	1.05	0.92
AP urinary tract	1.01	N/A	N/A	1.13

N/A means that the phantom in the denominator is not appropriate for that examination.

The reproducibility and the linearity of the output were better than 10% (typically, about 5%) for all radiographic systems. The kVp accuracy was ±3% for all measurements.

In Table 4 (fourth column) the accuracies $A_{\text{ALD}},A_{\text{CHE}},A_{\text{SKU}},A_{\text{UNI}}$, and $A_{A31}$ of the mathematical model relative to the other phantoms are given, based on Table 1. In this case, the BSF value used is 1.35. The averages of all accuracies are also provided to show how different phantoms compare in general, even though not all phantoms are suitable for all examinations.

**Table 4:** Accuracies of the mathematical model relative to the other phantoms based on Table 1: $A_{\text{ALD}}$, $A_{\text{CHE}}$, $A_{\text{SKU}}$, $A_{\text{UNI}}$, and $A_{A31}$ with BSF=1.35; ${}^{\text{NEW}}A_{\text{ALD}}$, ${}^{\text{NEW}}A_{\text{CHE}}$, ${}^{\text{NEW}}A_{\text{SKU}}$, ${}^{\text{NEW}}A_{\text{UNI}}$, and ${}^{\text{NEW}}A_{A31}$ recalculated using the BSF from previous works^(15)^ ^,^ ^(16)^

(a) Alderson Rando phantom Examination	Number of radiological systems	Number of measurements	$A_{\text{ALD}}$	${}^{\text{NEW}}A_{\text{ALD}}$
PA skull	4	5	0.07	0.06
LAT skull	4	5	0.25	0.24
AP chest	5	9	0.25	0.22
PA chest	4	5	0.01	0.01
LAT chest	4	5	0.01	0.01
AP lumbar spine	4	5	0.07	0.07
LAT lumbar spine	4	5	0.09	0.09
LAT lumbo‐sacral joint	4	5	0.09	0.09
AP abdomen	4	5	0.34	0.34
AP pelvis	5	7	0.23	0.23
AP urinary tract	4	6	0.34	0.34
Average			0.16	0.15

(b) Chest phantom Examination	Number of radiological systems	Number of measurements	$A_{\text{CHE}}$	${}^{\text{NEW}}A_{\text{CHE}}$
AP chest	13	16	0.15	0.14
PA chest	10	13	0.16	0.15
LAT chest	10	13	0.13	0.13
Average			0.15	0.14

(c) Skull phantom Examination	Number of radiological systems	Number of measurements	$A_{\text{SKU}}$	${}^{\text{NEW}}A_{\text{SKU}}$
PA skull	15	18	0.10	0.08
LAT skull	18	20	0.08	0.06
Average			0.09	0.07

(d) Uniform phantom Examination	Number of radiological systems	Number of measurements	$A_{\text{UNI}}$	${}^{\text{NEW}}A_{\text{UNI}}$
PA skull	8	10	0.12	0.12
LAT skull	8	9	0.16	0.14
AP chest	5	7	0.23	0.22
PA chest	5	7	0.07	0.07
LAT chest	5	6	0.06	0.06
AP lumbar spine	16	18	0.10	0.10
LAT lumbar spine	16	17	0.13	0.12
LAT lumbo‐sacral joint	11	12	0.17	0.16
AP abdomen	24	30	0.11	0.10
AP pelvis	24	30	0.13	0.13
AP urinary tract	22	25	0.10	0.10
Average			0.13	0.12

(e) AAPM31 phantom Examination	Number of radiological systems	Number of measurements	$A_{A31}$	${}^{\text{NEW}}A_{A31}$
PA skull	6	7	0.19	0.15
LAT skull	5	6	0.25	0.19
AP chest	4	5	0.05	0.05
PA chest	3	4	0.07	0.02
LAT chest	3	4	0.08	0.01
AP lumbar spine	10	12	0.07	0.07
LAT lumbar spine	6	8	0.08	0.06
LAT lumbo‐sacral joint	7	9	0.09	0.07
AP abdomen	11	16	0.15	0.14
AP pelvis	8	11	0.12	0.11
AP urinary tract	9	13	0.15	0.14
Average			0.12	0.09

**Figure 2:**
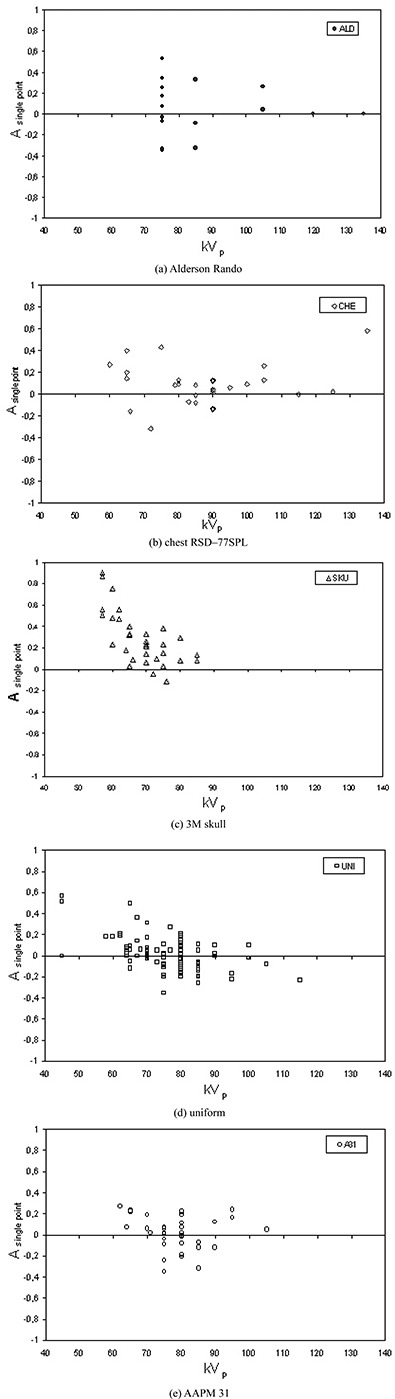
Mathematical model accuracy $A_{\text{singlepoint}}$ relative to the anthropomorphic and physical phantoms: (a) Alderson Rando; (b) chest RSD−77SPL (c) 3M skull; (d) uniform; (e) AAPM 31.

The $A_{\text{OVE}}$ is better than 14%. This value is of the same order reported in other works.^(11)^ ^,^ ^(13)^ ^,^ ^(14)^ Figures 2(a) to (e) show the accuracies $A_{\text{singlepoint}}$ of the mathematical model relative to the other phantoms plotted as a function of the kVp used. Using Eq. (3) to calculate this parameter should better visualize and take into account the displacements around the 0 value (which would mean perfect correspondence between phantoms).

Total mathematical model uncertainty, estimated on the order of ±12%, was calculated by quadratic propagation of errors of the single model components, that is, using only one BSF and the possible variation in X‐ray tube output owing to the time elapsed from the latter quality control. Total ionization chamber uncertainty, calculated in the same way, was estimated to be of the order of ±3% due to both the calibration factor given by the SIT center and the experimental FSD measurements. The use of only one BSF value was the most significant source of error. The idea of reducing it to a wider set of BSF values has been adopted.

Many papers have been published regarding the calculated or measured BSF.^(^ ^15^ ^–^ ^21^ ^)^ Listed in the fifth column of Table 4 are the new accuracies and their averages relative to the anthropomorphic and physical phantoms (${}^{\text{NEW}}A_{\text{ALD}}$, ${}^{\text{NEW}}A_{\text{CHE}}$, ${}^{\text{NEW}}A_{\text{SKU}}$, ${}^{\text{NEW}}A_{\text{UNI}}$, and ${}^{\text{NEW}}A_{A31}$) recalculated using the BSF from Harrison^(15)^ and Grosswendt,^(16)^ which are listed as a function of both the field size in the various examinations and the kVp values used. The ${}^{\text{NEW}}A_{\text{OVE}}$ has now improved, better than 12%.

## IV. DISCUSSION

The backscatter factor for a simple water phantom can be written as

(5) $${\text{BSF}}_{W}^{\left(W\right)}=\frac{X^{\left(W\right)}{\left[\frac{\mu_{\text{tr}}}{\rho }\right]}_{w,\text{air}}^{\left(W\right)}}{X^{\left(\text{free}\right)}{\left[\frac{\mu_{\text{tr}}}{\rho }\right]}_{w,\text{air}}^{\left(\text{free}\right)}}$$

where $X^{\left(w\right)}$ is the exposure at the surface of the water phantom, $X^{\left(\text{free}\right)}$ is the exposure at the same point in space without the phantom, and ${{[\mu }_{\text{tr}}/\rho ]}_{w,\text{air}}^{\left(w\right)},{\left[\mu_{\text{tr}}/\rho \right]}_{w,\;\text{air}}^{\left(\text{free}\right)}$ are the ratios of the mass‐energy transfer coefficients for water and air in the presence of scatter medium and in free space, respectively.

In Eq. (5), the ratios of the energy transfer coefficients are for the same media, but they cannot be cancelled because they are determined for different photon spectra: ${\left[\mu_{\text{tr}}/\rho \right]}_{w,\;\text{air}}^{\left(\text{free}\right)}$ is averaged over the spectral energy fluence distribution of the beam at the phantom surface, and ${\left[\mu_{\text{tr}}/\rho \right]}_{w,\;\text{air}}^{\left(\text{free}\right)}$ is averaged over the spectral energy fluence distribution of the primary beam without the phantom. If phantoms different from water are used, an analogous relationship of course can be applied. These theoretical considerations can explain why the backscatter factors and the ESDs are very strongly dependent on the material, shape, and size of the different phantoms.

Three of six phantoms used in this study are anthropomorphic. The ALD phantom is used more in radiotherapy, but it can be used at diagnostic energies as well.^(22)^ The CHE and SKU phantoms are more useful in radiology because they are optimized for image quality studies. The two physical phantoms can be more easily available than the anthropomorphic phantoms, but the latter better simulates the full scatter properties of human tissue. The ALD, CHE, and SKU phantoms have soft‐tissue‐equivalent material, which almost exactly duplicates water (in radio‐absorptive and scatter properties), and synthetic skeletons with cortical bone, which is radio‐equivalent to natural bone and matches the volumetric electron densities and the mass attenuation coefficients of ICRU‐44^(23)^ across the entire energy range of diagnostic energies. It is therefore interesting to select for every examination an anthropomorphic phantom to be used as a “gold standard” relative to the physical phantoms and to analyze the physical versus anthropomorphic phantoms comparisons.

The gold standard for the PA chest, AP chest, and LAT chest is the CHE phantom. In every measurement it gives lower ESDs than physical phantoms. A possible explanation for this result is that the backscattered radiation is less when lungs are involved, and the nonanthropomorphic phantoms are not able to take into account this physical process. Moreover, when automatic exposure controls (AECs) are used, the phantoms can have a significantly different “equivalent thickness”^(24)^ and, therefore, a different ESD value. In AEC systems we investigated, there are usually three detectors located as the vertices of a triangle. Every detector can be used alone or together with the others, depending on the examination. For example, in PA chest the two lateral detectors are used; in LAT chest the central detector is used; and in AP pelvis all three detectors are usually used. Therefore, in chest examinations, if in AEC systems lung‐centered detectors are used, the physical phantoms, which are soft‐tissue‐equivalent in terms of primary transmission, will transmit less radiation to a “lung‐field detector” than would anthropomorphic phantoms, and the AEC will terminate exposure too late. This effect will lead to (ESD physical phantoms)/(ESD CHE) ratios being higher than one for chest examinations in Table 3.

The reference for PA skull and LAT skull is the SKU phantom. In every measurement it gives lower ESDs than physical phantoms. In Figs. 2(a) to (e) at lower kVp values there is a large spread of the $A_{\text{singlepoint}}$ values because the dependence of ESDs on different atomic numbers of phantoms is stressed. The kVp values used in clinical practice for skull examinations are the lowest in all examinations considered. At these low energies the interactions of photons in the phantoms are modulated differently by the atomic numbers of soft tissue and bone. In the case of soft tissue, the Compton effect predominates; in the case of bone, the Compton and photoelectric effects are approximately equivalent.^(25)^ For this reason, the backscattered radiation is more in the soft tissue than in the bone. Therefore, the ESD in physical phantoms (almost entirely made in PMMA) is higher than in SKU phantoms (made of rubber and bone equivalent). Another factor that affects the different responses is this: a rounded surface such as that on the SKU phantom reduces the amount of scattered radiation, contributing to ESD.

For the remaining examinations (AP abdomen, AP lumbar spine, LAT lumbar spine, LAT lumbo‐sacral joint, AP pelvis, and AP urinary tract), the gold standard is the Alderson Rando phantom: it gives (except two cases) higher ESDs than physical phantoms. A possible explanation is that in these examinations, where the energies are higher, the more accurate composition of anthropomorphic phantoms takes better into account the higher backscatter radiation. Between the two physical phantoms, A31 is slightly better because of its structure, more sophisticated relatively to the simpler uniform phantom.

Note in Table 3 that, in general, for every couple of phantoms, the REFs Phantom1/Phantom2 are not always equal to the inverse of the corresponding REFs Phantom2/Phantom1. The explanation is that the REFs reported in Table 3 are averages of ratios of ESDs measured on many different radiological systems, where the same kind of examination is performed with different technique factors, and the phantom's response is nonlinear at different energies. From a strictly theoretical point of view, this is a limitation because all the examinations should be performed with a clinical protocol that gives exactly the same ESD to the standard patient. However, from a more practical and realistic point of view, that aim is seldom reached clinically because it is dependent on radiological equipment characteristics as well as the working procedures of the operators. Therefore, the above‐reported “theoretical limitation” becomes an interesting “working hypothesis.” The number of radiological systems, reported in Table 1, includes a wide range of equipment typologies used in clinical practice, and, together with the high number of measurements made, it should give a consistent reliability in all operative clinical conditions to comparisons presented in this paper.

Since the individual radiographic systems had reproducibility and linearity values better than 5%, the variability of ESD and then of REF values is most likely attributable to variations in phantom material, design, and characteristics, which are exactly the distinctive features to study.

As far as the mathematical phantom is concerned, the model has been validated by more than 400 ESD calculations compared with measurements taken on different phantoms and radiological equipment. An overall accuracy better than 12% was obtained, comparable with data reported in literature. Therefore, in ESD calculations, this model can be used satisfactorily because it presents many advantages. First, it allows the optimization of available resources by using data already taken during quality controls. Second, the ESDs are calculated theoretically with a general formula but are also based on direct measurements, so that every radiological system keeps its own characteristics. Finally, the model is very easy to implement using an electronic spreadsheet and to use as a dynamic tool.

## V. CONCLUSIONS

It is difficult to make comparisons between patient dose results in different studies because different measuring phantoms are used. In this respect, the comparison performed in the present study could be useful in order to have data comparable with those taken both at different times and with different phantoms, in the same or in other radiology departments. In this way, the phantom used most often for ESD measurements in diagnostic radiology—the uniform phantom—can be compared with other more sophisticated but less available phantoms, and each of them can be compared with a simple and easy‐to‐use mathematical model.

This study shows that in measuring ESD values, the phantoms are not as “standard” as the medical physicist wishes, but that it is possible to take into account the relative differences in order to have more comparable and consistent data.

## ACKNOWLEDGMENTS

We wish to express our thanks to Marina Benati and Luciano Selleri for their assistance in data collection.
